# Continuous Monitoring Enables Dynamic Biomarkers to Assess Resilience in Acute COVID-19 Patients

**DOI:** 10.3390/jcm14030951

**Published:** 2025-02-02

**Authors:** Jerrald L. Rector, Anna Kuranova, Marcel G. M. Olde Rikkert, Harry van Goor, René J. F. Melis, Sebastian J. H. Bredie

**Affiliations:** 1 Department of Geriatric Medicine, Radboud University Medical Center, 6525 GA Nijmegen, The Netherlands; jerraldrector@hotmail.com (J.L.R.); marcel.olderikkert@radboudumc.nl (M.G.M.O.R.); rene.melis@radboudumc.nl (R.J.F.M.); 2 Department of Surgery, Radboud University Medical Center, 6525 GA Nijmegen, The Netherlands; harry.vangoor@radboudumc.nl; 3 Department of Internal Medicine, Radboud University Medical Center, 6525 GA Nijmegen, The Netherlands; bas.bredie@radboudumc.nl

**Keywords:** oxygen saturation, vital signs, biomarkers, COVID-19, oxygen inhalation therapy

## Abstract

**Background/Objectives**: The effective management of acute illnesses like COVID-19 requires tools to dynamically assess a patient’s resilience to health stressors. This study evaluates novel dynamic biomarkers from continuous blood oxygen saturation (SpO_2_) monitoring, exploring their association with patient outcomes to support clinical decision making. **Methods**: We examined 200 hospital admissions from 181 adults diagnosed with COVID-19. Two dynamic biomarkers reflecting the homeostatic regulation efficiency of SpO_2_ were developed to assess their association with adverse hospital outcomes, specifically ICU admission or death, using binary logistic regressions. The resilience exponent α recorded the relative frequency of prolonged SpO_2_ declines, while O_2_ challenges quantified the dynamic response to changes in O_2_ supplementation. **Results**: Increased resilience exponent α corresponded to decreased odds of adverse outcomes (OR [95% CI] = 0.59 [0.37–0.93], *p* = 0.03). Larger SpO_2_ increases in response to O_2_ supplementation were associated with increased odds of adverse outcomes (OR [95% CI] = 1.40 [1.04–1.83], *p* = 0.03). Additionally, the number of O_2_ supplementation increases (OR [95% CI] = 2.91 [1.90–4.49]) and decreases (OR [95% CI] = 0.33 [0.20–0.55]) during hospitalization were independently linked to poorer and improved outcomes, respectively (both *p* < 0.001). **Conclusions**: The resilience exponent α and the O_2_ challenge response provide insights into the dynamic regulation of SpO_2_, reflecting physical resilience in COVID-19 patients. Continuous SpO_2_ monitoring in acute care settings could support more informed clinical decisions during patient management.

## 1. Introduction

Physical resilience—the dynamic ability to resist and recover from health perturbations—can be recognized as a decisive prognostic factor in patient care [1,2]. Assessing a patient’s resilience upon admission and monitoring changes throughout hospitalization can inform decision making and identify deterioration before adverse events occur.

Traditional approaches to understanding resilience, often implicitly inferred from static factors such as age, sex, body mass index (BMI), and medical history, can overlook its dynamic nature. This study introduces a novel method that focuses on patients’ dynamic responses over time to assess their ability to ‘bounce back’. This dynamic approach offers a new lens to study and understand resilience, potentially providing more informative insights than traditional methods. Resilience is inherently dynamic, involving multiple subsystems that interactively shape responses to stressors. Observing the resistance to and recovery from health challenges at sufficient temporal resolution can reveal the quality of these regulatory mechanisms [3,4].

Vital signs like core temperature are optimal when maintained within narrow bounds around a homeostatic equilibrium. Fluctuations in these regulated variables indicate the functioning of underlying regulatory mechanisms striving to restore homeostasis [5]. Here, we derive new quantitative metrics capturing these homeostatic deviations, considering them as dynamic biomarkers reflecting a patient’s ability to resist dysregulation due to disease and, by extension, their resilience. Unlike traditional static measures, these biomarkers leverage continuous monitoring data to provide real-time, dynamic insights into pulmonary regulation, offering potential applications for predictive modeling and clinical decision making in acute care settings.

In COVID-19 patients, diminished physical resilience may hold significant prognostic value that can potentially be used to decide on transitions to different levels of care [6]. However, dynamic biomarkers quantifying this resilience are lacking. The key vital signs to monitor in COVID-19 include respiratory rate (RR), heart rate (HR), and peripheral blood oxygen saturation (SpO_2_). SpO_2_ is uniquely suitable for continuous and non-invasive measurement, reflecting pulmonary homeostasis in COVID-19 patients [7,8].

COVID-19-induced pulmonary distress presents a persistent homeostatic challenge. The sustained resistance to and recovery from SpO_2_ drops may represent patients’ resilience. Typically, drops in SpO_2_ trigger mechanisms (e.g., increased RR) that quickly restore equilibrium [9]. Prolonged declines indicate a failure of these mechanisms. Responses to O_2_ supplementation can serve as semi-standardized stimulus–response tests assessing SpO_2_ regulation resilience [10]. We hypothesize that more resilient patients will regain higher SpO_2_ levels more quickly upon receiving O_2_ supplementation and exhibit smaller declines in SpO_2_ when O_2_ is gradually removed.

This study leverages data from 181 unique individuals accounting for 200 admissions with Reverse Transcription Polymerase Chain Reaction (RT-PCR)-confirmed COVID-19, including continuous vital sign monitoring data and demographic and clinical variables such as age, sex, and BMI. We developed two novel resilience features based on SpO_2_ data: the resilience exponent α and the O_2_ challenge response. The resilience exponent α quantifies the relative duration of SpO_2_ declines, indicating regulatory capacity. The O_2_ challenge response measures the SpO_2_ reaction to O_2_ supplementation adjustments. These biomarkers were compared between patients with poor outcomes (Intensive Care Unit (ICU) transfer or death) and others (e.g., discharge).

We hypothesize that patients with lower physical resilience, leading to ICU transfer or death, will show (1) frequent long-duration declines in SpO_2_ and (2) less favorable changes in SpO_2_ in response to O_2_ supplementation adjustments. In the context of the current study, a less favorable outcome is defined as the occurrence of ICU transfer or in-hospital death. Specifically, diminished resilience should result in smaller pre-post differences in SpO_2_ in response to O_2_ increases and larger drops in SpO_2_ with O_2_ withdrawal. This study aims to develop and validate dynamic biomarkers based on SpO_2_ regulation in COVID-19 patients to predict clinical outcomes, particularly ICU transfer or in-hospital death.

## 2. Materials and Methods

### 2.1. Participants and Clinical Data

This study encompasses data from 200 admissions of RT-PCR-confirmed COVID-19 patients to a Dutch academic hospital between March and September 2020. These admissions correspond to 181 unique individuals: 163 patients were admitted once, 17 were admitted twice, and 1 was admitted thrice. Standardized data per admission, such as age, sex, and BMI, were anonymized and chronologically recorded in an electronic case record form, Castor E.D.C., Ciwit BV, Amsterdam, The Netherlands.

The continuous monitoring of vital signs was conducted as a part of standard care in general nursing wards (e.g., geriatrics, surgery, and pulmonology). These wards are non-intensive care settings, and standardized monitoring protocols were applied uniformly across all the patients using the ViSi Mobile system (Sotera Wireless, San Diego, CA, USA). This wearable device recorded parameters such as heart rate (HR), respiratory rate (RR), peripheral oxygen saturation (SpO_2_), skin temperature, and cuffless blood pressure at one-minute intervals [11]. All data were automatically stored in our research database, and each admission was treated as an independent case for data analysis. Data processing, feature extraction, and statistical analysis were performed using MATLAB [12].

The research use of monitoring data was approved on 23 April 2018 by the Radboudumc Medical Ethics Committee (continuous monitoring with predictive analysis in the unsupervised ward to improve clinical outcomes: 2018-4330) and conducted in line with the Helsinki Declaration. By Dutch law, medical record data can be retrospectively used upon informing patients that they can opt out at any time.

Admission outcomes were categorized into ‘poor’ and ‘other’. The ‘poor’ outcome was characterized by either a transfer to the ICU or death. This combination of outcomes has been frequently used in many of the prognostic models identified in a systematic review [13]. The ‘other’ outcome included patients either discharged or transferred to a different ward before eventual discharge [14]. To study the impact of resilience on the likelihood of poor outcomes, a binary outcome variable was utilized (poor = 1; other = 0).

### 2.2. Resilience Features

#### 2.2.1. Resilience Exponent α

**Figure 1:**
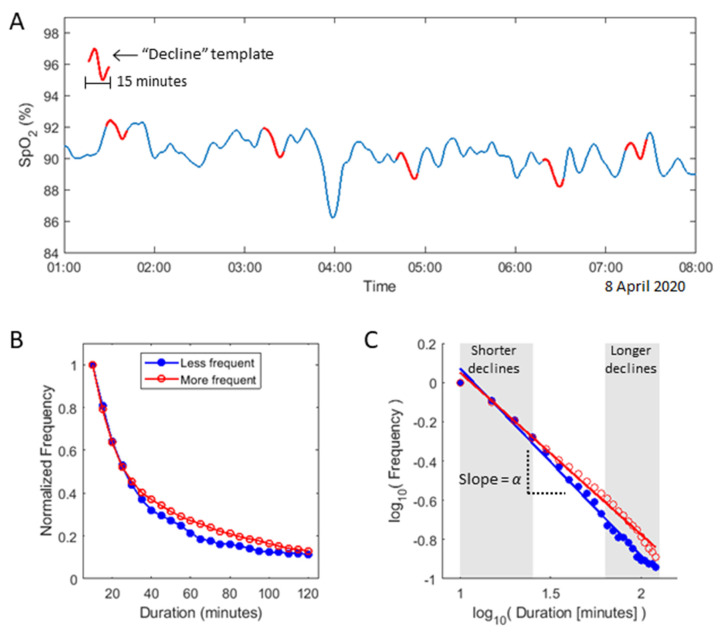
Derivation of the resilience exponent α from SpO_2_ decline data. (**A**) The pre-processed SpO_2_ signal is convolved with 23 distinct ‘decline’ templates, each representing a SpO_2_ drop of a particular duration, ranging from 10 to 120 min. An example of a 15 min ‘decline’ template is illustrated. (**B**) The frequency of matches for each decline template (i.e., the number of times a given duration of SpO_2_ drop is observed) throughout the patient’s entire hospitalization is plotted against the decline’s duration. (**C**) The plotted points reveal a linear relationship when viewed on a log-log scale, with the slope of the line of best fit representing the resilience exponent α. The exponent α measures the relative frequency of long-duration to short-duration SpO_2_ declines. Flatter slopes (α values closer to 0) suggest a preponderance of long-duration SpO_2_ declines, indicating less resilience. Conversely, steeper slopes (higher α values) indicate fewer long-duration declines, signifying greater resilience. For comparison, panels B and C show data from two patients: one in red with more frequent long-duration declines and one in blue with fewer.

To capture the dynamics of SpO_2_ regulation over a patient’s hospital stay, we constructed a novel resilience feature, the resilience exponent α. This feature quantifies the relative frequency of declines in SpO_2_ of different durations, reflecting the patient’s ability to recover from disturbances to homeostasis. Specifically, the resilience exponent α is derived from the observed frequency distribution of SpO_2_ decline durations, which we hypothesized to follow a power-law relationship of form F(x) ∝ x^(−α). Here, F(x) represents the observed frequency of declines lasting x minutes, and α is a scaling parameter. Under this model, a higher α value indicates fewer prolonged declines in SpO_2_ relative to short declines, suggesting better regulatory capacity and resilience. Conversely, a lower α value, indicating a higher relative frequency of prolonged SpO_2_ declines, suggests poorer regulation and resilience (see Figure 1). The detailed methodology for calculating the resilience exponent α is provided in the Supplementary Materials.

#### 2.2.2. The O_2_ Challenge Response

**Figure 2:**
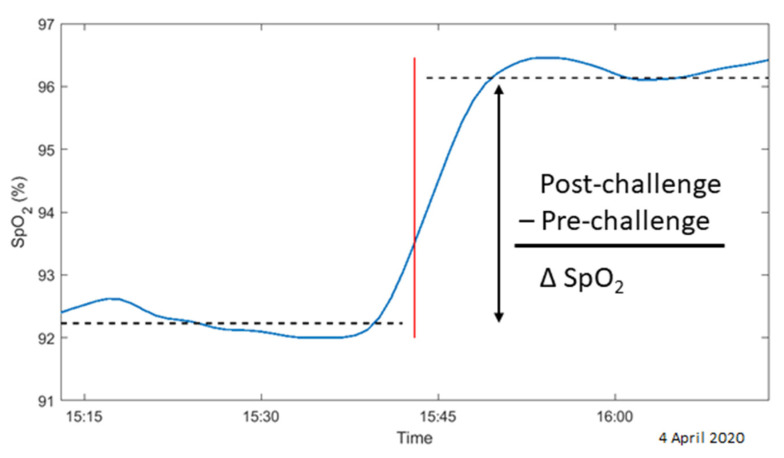
Extraction of ΔSpO_2_ (vertical black arrow) from O_2_ challenge data. For each alteration in O_2_ supplementation (marked by the red vertical line), the average SpO_2_ level (depicted by the blue line) was calculated over two 30 min windows: one immediately before and the other immediately after the change (indicated by the dashed black lines). The ΔSpO_2_ was then determined as the difference between the post-challenge and pre-challenge SpO_2_ averages.

Secondly, we assessed the patient’s SpO_2_ responses to alterations in O_2_ supplementation, termed the “O_2_ challenge response”. This approach borrows from the stimulus–response paradigm, wherein a system or subsystem’s parameter is perturbed using a standardized stimulus, and the system’s response is meticulously observed over time. This response—specifically, its resistance and recovery—estimates the system’s resilience [10]. In this study, we treated adjustments to O_2_ supplementation as semi-standardized stimuli, probing the resilience of SpO_2_ homeostasis. The more robust the SpO_2_ level response to these adjustments, the higher the resilience. The change in SpO_2_ (ΔSpO_2_) in response to an O_2_ supplementation adjustment was quantified as the mean difference in SpO_2_ over a 30 min window immediately preceding and following the adjustment (see Figure 2). Details regarding the calculation of ΔSpO_2_ can be found in the Supplementary Materials, Document S1.

### 2.3. Statistical Analyses

We started our statistical analysis by describing the baseline and clinical characteristics of the two outcome groups. The variables were described using mean and standard deviations (or median and interquartile range for non-normally distributed variables) or proportions. The statistical testing of differences between the groups was conducted using the appropriate test statistic depending on measurement type and the distribution of the data (the exact statistics used are in the footnote of Table 1). Then, we continued with a binary logistic regression to compare the newly defined resilience features between the two outcome groups. Two separate analyses were performed for each resilience feature.

In the first analysis, the resilience exponent α was the independent predictor in an unadjusted model (Model 1). Subsequently, this model was adjusted for age, sex, and BMI (Model 2), and the mean SpO_2_ recorded throughout the hospitalization period (Model 3).

The second analysis focused on the O_2_ challenge responses and followed the same structure as Models 1 to 3 above, except that the mean ΔSpO_2_ for O_2_ increases and the mean ΔSpO_2_ for O_2_ decreases were simultaneously introduced as two independent predictors. An additional model (Model 4) further adjusted for the number of O_2_ changes for both increases and decreases.

It is important to note that for each patient with multiple admissions, these admissions were treated as independent in the analysis. As such, some individuals contributed more than one set of resilience features to the overall dataset, which should be considered when interpreting the results. The decision to treat each admission independently was made because the resilience features we examined—the resilience exponent α and the O_2_ challenge response—can potentially vary from one admission to another for the same patient. Therefore, each admission offers unique information about the patient’s physiological response to COVID-19, contributing to our understanding of the diverse manifestations of this disease.

This adjusted approach to the analysis was designed to provide a more comprehensive understanding of the potential predictors of outcomes in COVID-19 patients, particularly concerning their physical resilience and responses to O_2_ supplementation adjustments.

## 3. Results

### 3.1. Sample Characteristics

Characteristics of the 200 admissions from 181 unique patients are detailed in Table 1. Of these admissions, 148 (74%) ended with the patients being discharged, while 16 (8%) were instances where the patients were transferred to another ward before ultimately being discharged. In 20 cases (10%), the patients were transferred to a guarded (intensive care) ward, and 15 (8%) ended in death. For unknown reasons, monitoring was prematurely stopped for one admission (0.5%). A total of 35 admissions (17%) were classified as ‘poor’ outcomes, while the remaining 165 (83%) were categorized as ‘other’ outcomes.

The median patient age for these admissions was 65 years (IQR = 17), with females constituting 34.5% of the admissions and a mean BMI of 27 (SD = 5) kg/m^2^. The median of the mean SpO_2_ across hospitalizations was 95% (IQR = 1.7%). The only patient characteristic that significantly differed between the outcome groups was age, with the poor outcome group having a median age of 67 years versus 64 years in the other outcome group (*p* = 0.050).

The admissions characterized as poor outcomes required more frequent increases in O_2_ supplementation (median [IQR] = 5 [3] versus 2 [3]) and had fewer decreases in O_2_ (median [IQR] = 2 [2.8] versus 3 [3], both *p* < 0.01) compared to the other outcome admissions. The SpO_2_ immediately preceding increases in O_2_ supplementation tended to be lower for the admissions classified as poor outcomes (93% vs. 94%; *p* < 0.10).

### 3.2. Resilience Exponent α and Outcomes

The resilience exponent α was lower in the poor outcome group compared to the other outcome group (1.815 vs. 1.822, *p* = 0.04), suggesting more frequent long-duration declines in SpO_2_. The average ΔSpO_2_ in response to O_2_ increases in the poor outcome group was double that of the other outcome group (1.02% ΔSpO_2_ vs. 0.47%, respectively, *p* = 0.10), reflecting a tendency of larger ΔSpO_2_ in response to O_2_ increases. There were no significant differences in ΔSpO_2_ between the two outcome groups in response to O2 decreases (*p* = 0.38).

Table 2 shows the influence of the exponent α on the odds of a poor outcome. Model 1 shows that a one-SD increase in α was associated with a 36% reduced odds of poor outcome (Odds Ratio (OR) [95% Confidence Interval, CI] = 0.64 [0.42–0.98], *p* = 0.047). After adjustment for age, sex, BMI, and mean SpO_2_, this effect of α increased to 41% (Model 3: OR [95% CI] = 0.59 [0.37–0.93], *p* = 0.03). Additionally, in Model 3, a 1% higher mean SpO_2_ was associated with a 29% reduced odds of poor outcome (OR [95% CI] = 0.71 [0.56–0.90], *p* = 0.005). None of the other static measures (i.e., age, sex, or BMI) were significantly associated with hospitalization outcomes.

### 3.3. O_2_ Challenges and Outcomes

Table 3 shows the results of the O_2_ challenge analyses. After adjustment for age, sex, and BMI (Model 2), every 1% increase in the average ΔSpO_2_ in response to O_2_ increase was associated with a 36% increased odds of poor outcome (OR [95% CI] = 1.36 [1.03–1.80], *p* = 0.03). This effect remained after additional adjustment for mean SpO_2_ (40%, *p* = 0.03; Model 3).

Model 4 included the number of O_2_ increases and decreases separately as covariates. In this final model, each additional O_2_ increase and decrease was associated with a nearly 3-fold increase and a 67% reduction in the odds of poor outcome, respectively (increases: OR [95% CI] = 2.91 [1.90–4.49]; decreases: OR [95% CI] = 0.33 [0.20–0.55]; both *p* < 0.001). For all the models, neither the static measures (i.e., age, sex, BMI, and mean SpO_2_) nor the ΔSpO_2_ in response to O_2_ decreases were significantly associated with the outcome.

## 4. Discussion

Our research investigated the association between dynamic resilience biomarkers derived from continuous SpO_2_ monitoring and poor hospital outcomes in COVID-19 patients. Notably, these outcomes were analyzed at the level of hospital admissions rather than individual patients, with some patients having multiple admissions. This approach allowed us to track variations in the patient’s physiological responses with greater accuracy, capturing a dynamic view of health stability that may surpass the static, intermittent biomarkers currently in use.

The resilience exponent α, indicative of long-duration SpO_2_ declines during hospitalization, was associated with outcomes. Specifically, a higher α value, reflecting fewer SpO_2_ dips and greater resilience, corresponded to reduced odds of poor outcomes. Compared to the current methods of analyzing SpO_2_ data, which often focus on mean or extremal values, our exponent α captures the patient’s dynamic response to health stressors. This novel methodology, therefore, provides unique information, offering a potentially fuller perspective on physiological regulation.

In our study, the patients’ responses to oxygen supplementation challenges did not align with our initial hypothesis. We assumed that a larger increase in the SpO_2_ levels following supplemental oxygen administration—ostensibly a sign of the patient’s resilience—would correlate with better outcomes. However, we found that it was associated with increased odds of poor outcomes. This association was no longer significant after adjusting for the number of times oxygen supplementation was adjusted, which introduces some possible considerations for clinical decision-making processes.

Additionally, the frequency of oxygen adjustments seemed to be more strongly associated with the outcome. More specifically, instances where oxygen supplementation was increased were associated with worse outcomes, while instances where oxygen was decreased suggested better recovery. A potential ceiling effect may influence these results. The patients requiring more frequent oxygen adjustments, likely starting from a lower baseline SpO_2_, would naturally have a more extensive “range” for SpO_2_ to increase. As a result, the observed larger increases in SpO_2_ might simply reflect this initial lower starting point rather than indicating a robust response to supplemental oxygen. Further analysis is needed to understand these dynamics fully. The use of aspects of oxygen saturation or supplementation to predict COVID-19 is part of some prognostic models [13]. Our findings also add to earlier studies that studied the predictive value of post-exertional saturation changes [15,16], which fits with the stimulus–response paradigm [10] from which we developed the oxygen supplementation challenge measure. So far, both approaches to challenge the respiratory system and derive meaningful associations with outcomes from this have had modest results.

Our study contributes to the knowledge of physical resilience by demonstrating the association of dynamic biomarkers obtained from continuous vital sign data with hospital outcomes. This aligns with earlier research that used complex systems resilience principles to differentiate between healthy and diseased states, like frailty, cardiovascular disease, and Parkinson’s [17,18,19,20]. Additional research suggested that the indicators of resilience, particularly those of critical slowing down, provide valuable insights in critical care settings [21,22].

Using these concepts as a foundation, we developed a unique measure: the resilience exponent α. This measure utilizes a power-law relationship to quantify the duration of SpO_2_ declines during a patient’s hospital stay. A previously developed dynamic complexity metric in complex systems foreshadows imminent critical transitions by identifying large fluctuations and high state occupancy [23,24]. These fluctuations are akin to our resilience exponent α, in that frequent long-duration SpO_2_ declines could indicate an upcoming instability, potentially signaling adverse hospitalization outcomes. Our exponent α can be viewed as a practical application of critical slowing down principles in patient care, offering potential early warnings of health deterioration.

Utilizing the resilience exponent α in the context of COVID-19 may be especially relevant, as SpO_2_ is a critical prognostic factor for the infection’s outcomes. In this study, rather than just summarizing vital signs by comparing averages or trends across the monitoring period [25,26], we introduced biomarkers that offer insights into the active homeostatic regulation of SpO_2_. This shift from a derangement-based to a resilience-focused paradigm augments our understanding of resilience in critical health scenarios and demonstrates how novel methods can potentially be applied to enhance patient management in hospital settings.

### Strengths and Limitations

Our study has several notable strengths. Firstly, it introduces a unique perspective on resilience, representing a departure from the commonly utilized deficit-based assessments such as frailty. Secondly, our utilization of continuous vital sign monitoring allowed us to exploit high-quality data and established protocols implemented in our hospital well before the onset of the COVID-19 pandemic. Finally, our cohort’s significant heterogeneity in initial health status and resilience was a rigorous test for the proposed dynamic biomarkers.

However, some limitations warrant attention. The first limitation arises from our decision to conduct analyses at the level of hospital admissions, not individual patients. This meant that some patients contributed multiple admissions to our analysis, potentially diluting the association of our biomarkers with the outcome. We cannot rule out the possibility that the association of our novel indicators with the outcome may differ between persons with first and readmissions. The sample size did not warrant separating the two groups in our analyses. This implies that if, in reality, the association differs considerably between persons upon first and readmissions, the current averaged association observed across both groups has limited generalizability. We suggest that the replication of our findings in larger samples that allow us to analyze these groups separately is important.

The calculation of the resilience exponent α also presented challenges. The α did not account for the initial SpO_2_ levels when assessing the duration of SpO_2_ declines. For instance, a 2% drop in SpO_2_ to 70% was treated equivalently to a 2% drop to 80%. Although this may have been partially addressed by adjusting for mean SpO_2_, future work could consider weighting each duration based on pre-decline SpO_2_ for greater accuracy. Furthermore, α did not differentiate between SpO_2_ dips that self-recovered and those necessitating supplemental oxygen, which could have confounded the interpretation of resilience. Also, the resilience exponent α may have depended on the response of the medical staff to declines in SpO_2_. Despite the fact that all the participants were monitored continuously using the same monitoring and staff response protocols, given that our study was conducted in routine clinical practice, heterogeneity in the response of medical staff may have occurred. As medical staff interventions will likely have minimized the excursions in SpO_2,_ we expect any resulting bias to be towards nil.

Another limitation is related to the O_2_ challenge biomarker. Several methods were used to deliver supplemental oxygen, complicating direct comparisons of responses. Furthermore, the time of changes in O_2_ supplementation was retrospectively recorded, leading to potential misalignment with the SpO_2_ signal and impacting the accuracy of ΔSpO_2_ values. Also, information on the amount of change could have improved the quality of our analyses.

The study’s timing during high hospital and ICU occupancy due to COVID-19 surges could also have influenced the outcomes. While this study was conducted in general nursing wards (e.g., geriatrics, surgery, and pulmonology), ICU admissions and resource limitations at the hospital may have indirectly impacted the composition of the patient population included in the study. For instance, the most severely ill patients who might have required ICU care could have remained in general wards due to limited ICU capacity during the pandemic. This complexity of real-world decision making may limit the generalizability of our findings to non-pandemic circumstances or different clinical settings. Additionally, our study lacked data on COVID-19 variants, limiting the analysis of their impact on disease severity and oxygenation. Corticosteroids were not used in the general nursing wards, and oxygen supplementation was provided via nasal cannulae or non-rebreathing masks, with volumes adjusted based on clinical judgment rather than standardized protocols. These factors reflect early pandemic practices and may limit generalizability to other contexts.

Another reason to interpret these findings cautiously is that the sample size limited our ability to completely account for the role of possible confounds from other factors—some of which are more readily available in routine care—that may explain the predictive associations between our newly developed indicators and the outcomes, such as a clinical history of COPD or sleep patterns during the hospital stay. Examples in which spurious associations due to confounders resulted in low accuracy and generalizability have been reported for predictive models [27]. This underscores the importance of replication in other/larger datasets. At the same time, an uncritical selection of adjustment variables may result in an overfit model [28], and careless controlling for a confounder can remove relevant subject-specific information [29]. All this can—like residual confounding—result in low accuracy and generalizability. Of final importance is that confounding bias carries a different relevance in predictive than in etiological modeling, and the handling of confounders depends on the intended use. Fever is a good example. Most of the time, the relation between fever and prospective outcomes is not direct, but instead an indirect one, with disease severity as the common cause for both. Fever is still useful because it is an easy and accessible marker for disease severity which may otherwise be much more difficult to establish. In addition to the need for replication, the clinical relevance of the indicators we developed, relative to other information, still needs to be established. Recognizing all these aspects, we have tried to carefully balance the pros and cons of confounder handling in light of the predictive aim of our study.

Finally, conducting the study in an academic hospital setting may have influenced the results due to the specific patient population and clinicians’ familiarity with continuous vital sign monitoring. Thus, our findings may not be fully generalized to other settings.

Despite these limitations, the potential of dynamic biomarkers as valuable additions to predicting patient outcomes is clear. However, further prospective studies in diverse settings and patient groups (e.g., those with and without thromboembolic complications) are needed to replicate these results, refine and validate these biomarkers, assess their real-time impact on patient outcomes, and gauge the effect of systemic pressures, such as hospital occupancy, on their predictive power. This can finally provide input for the systematic development, validation, and implementation of clinical prediction rules in which the new indicators proposed here may serve alongside other clinical information to make a valid and clinically feasible prediction that informs clinical decision making.

## 5. Conclusions

The resilience exponent α, as a dynamic biomarker, offers an innovative approach to assessing the robustness of homeostatic processes that regulate SpO_2_ fluctuations and return to equilibrium, thereby quantifying patient resilience. This marker was proposed under the stimulus–response paradigm; however, its practical application requires further refinement for successful implementation in clinical settings.

Our study suggests that alongside structural characteristics, extracting dynamic biomarkers from continuous vital sign monitoring presents a possible avenue for quantifying resilience in acute illness, offering an advance on the traditional derangement-based paradigm. This introduces a new perspective into biomedical informatics by moving beyond static data snapshots to understand the dynamics of physiological regulation.

As we navigate the uncharted waters of dynamic biomarkers, future prospective studies should focus on refining these markers and validating their applicability in diverse use cases. Importantly, their real-time impact on patient outcomes should be rigorously evaluated. Once fully developed, assessing and continuously monitoring patient resilience could have substantial implications for healthcare. Not only does it promise to improve patient outcome predictions, but it also has the potential to shape treatment decisions, suggesting a more patient-centered, resilience-focused approach to care.

This work marks a step in this direction, and we hope it encourages further investigations in this emerging field of resilience research.

## Figures and Tables

**Table 1:** Patient characteristics by hospitalization outcome.

Characteristics		Outcomes		
	Total (n = 200)	Poor (n = 35)	Other (n = 165)	*p*-Value
Age (years)	65 (17.0)	67 (14.5)	64 (18.3)	0.050
Female sex (n (%)) *^a^*	69 (34.5)	15 (42.9)	54 (32.7)	0.169
Body mass index (kg/m^2^) *^b^*	27.5 (5.4)	27.3 (5.8)	27.5 (5.3)	0.830
Mean SpO_2_ (%) *^c^*	95.2 (1.7)	94.9 (2.4)	95.2 (1.7)	0.078
Number of O_2_ changes *^d^*				
Increases	3 (4)	5 (3)	2 (3)	<0.001
Decreases	3 (4)	2 (2.8)	3 (3)	0.006
SpO_2_ pre-increase (%) *^e^*	93.7 (2.9)	93.0 (2.7)	94.0 (2.9)	0.097
SpO_2_ pre-decrease (%) *^e^*	95.3 (2.2)	95.2 (2.4)	95.3 (2.1)	0.693
Dynamic Biomarkers				
Exponent α *^b^*^,^*^f^*	1.821 (0.018)	1.815 (0.015)	1.822 (0.019)	0.037
Mean ΔSpO_2_ (%) *^g^*				
Increases	0.62 (1.64)	1.02 (1.64)	0.47 (1.63)	0.097
Decreases	−0.15 (1.07)	0.01 (1.40)	−0.19 (1.00)	0.381

Unless otherwise stated, the values are median (Interquartile Range, IQR) with *p*-value from the Mann–Whitney U test. *^a^* differences tested with the Χ2 test. *^b^* values are mean (standard deviation, SD) tested with the *t*-test. *^c^* average blood oxygen saturation (SpO_2_) across the entire hospitalization. *^d^* Number of O_2_ adjustments during hospitalization. *^e^* an average SpO_2_ before increases/decreases in O_2_ supplementation. *^f^* scaling exponent reflecting the relative frequency of shorter to longer declines in SpO_2_ (unitless) *^g^* average change in SpO_2_ in response to increases/decreases in O_2_ supplementation.

**Table 2:** Binary logistic regression for exponent α and poor hospitalization outcome.

Variable	Model 1	*p*-Value	Model 2	*p*-Value	Model 3	*p*-Value
Age (years)	--	--	1.03 (1.00–1.06)	0.072	1.02 (0.98–1.05)	0.37
Female sex	--	--	1.60 (0.73–3.47)	0.25	1.74 (0.77–3.91)	0.18
Body mass index (kg/m^2^)	--	--	0.99 (0.92–1.07)	0.83	0.98 (0.91–1.06)	0.60
Mean SpO_2_ *^a^*	--	--	--	--	0.71 (0.56–0.90)	0.0054
Exponent α *^b^*	0.64 (0.42–0.98)	0.038	0.64 (0.42–0.99)	0.047	0.59 (0.37–0.93)	0.025

Values are OR (95% CI). *^a^* an average SpO_2_ across the entire hospitalization; *^b^* semi-standardized (i.e., coefficients represent 1 SD increase in α).

**Table 3:** Binary logistic regression for O_2_ challenge features and ultimate hospital discharge.

Variable	Model 1	*p*-Value	Model 2	*p*-Value	Model 3	*p*-Value	Model 4	*p*-Value
Age (years)	--		1.01 (0.97–1.05)	0.58	1.01 (0.97–1.05)	0.73	1.05 (0.99–1.11)	0.11
Female sex	--		1.70 (0.67–4.33)	0.26	1.83 (0.71–4.72)	0.21	3.51 (0.87–14.20)	0.078
Body mass index (kg/m^2^)	--		1.00 (0.92–1.09)	0.93	1.00 (0.92–1.09)	0.95	0.99 (0.89–1.09)	0.79
Mean SpO_2_ *^a^*	--		--		0.85 (0.62–1.17)	0.32	0.97 (0.65–1.44)	0.87
Number of O_2_ increases	--		--		--		2.91 (1.90–4.49)	<0.001
Number of O_2_ decreases	--		--		--		0.33 (0.20–0.55)	<0.001
Mean ΔSpO_2_: increases	1.35 (1.03–1.79)	0.032	1.36 (1.03–1.80)	0.033	1.40 (1.04–1.83)	0.027	1.31 (0.86–1.99)	0.21
Mean ΔSpO_2_: decreases	1.29 (0.85–1.97)	0.23	1.26 (0.81–1.95)	0.3	1.27 (0.82–1.95)	0.29	0.93 (0.54–1.60)	0.79

Values are OR (95% CI). *^a^* an average SpO_2_ across the entire hospitalization.

## Data Availability

The datasets presented in this article are not readily available because they contain sensitive medical information and sharing them could compromise patient privacy. Requests to access the datasets should be directed to Sebastian Bredie (bas.bredie@radboudumc.nl) or Harry van Goor (harry.vangoor@radboudumc.nl). Access may be granted to qualified researchers subject to approval by the relevant ethics committee and the completion of a data use agreement.

## Supplementary Materials

The following supporting information can be downloaded at https://www.mdpi.com/article/10.3390/jcm14030951/s1, Document S1: Resilience COVID_SupMat [10,30].
