# Supplementary material for: Continuous Monitoring Enables Dynamic Biomarkers to Assess Resilience in Acute COVID-19 Patients
Source: J Clin Med. 2025 Feb 2;14(3):951. doi: 10.3390/jcm14030951 (PMC11818652; doi:10.3390/jcm14030951)
Supplement: Supplementary file 1 [file jcm-14-00951-s001.zip › Document S1. Resilience COVID_SupMat.pdf]

# Supplementary Information for “Continuous Monitoring Enables Dynamic Biomarkers to Assess Resilience in Acute COVID-19 Patients” by Rector, J.L. et al.

## Contents


Supplementary Materials and Methods ........ 2

Participants & clinical data ........ 2

Extracting resilience features ........ 2

Resilience exponent $\alpha$ ........ 2

$O_2$ challenge response ........ 3

References ........ 4

STROBE Checklist ........ 5

# Supplementary Methods

## Participants & clinical data

The study encompassed 200 admissions involving 181 unique COVID-19 patients at an academic medical center in the Netherlands between March and September 2020. Infection was confirmed using real-time reverse transcription polymerase chain reaction (RT-PCR) assays. Patients' potential COVID-19 pulmonary disease severity was assessed from a CT scan and admission was based on clinical judgement. The patients' clinical data during admission were anonymized and registered chronologically in an electronic case record form (Castor EDC, Ciwit BV, Amsterdam, NLD). Characteristics, such as age, gender, body mass index (BMI), and hospitalization outcome, were collected for all patients.

## Extracting resilience features

This study focused on two dynamic features obtained from the $SpO_2$ signal, either alone or in combination with routinely collected clinical information about $O_2$ supplementation, that help quantify resilience features of homeostatic functioning during the above-described clinical situation. First, the novel resilience exponent $\alpha$ was constructed as a global measure that captures the relative duration of observed drops in the patient's $SpO_2$ across their entire hospital stay. Second, the $O_2$ challenge response captures how the patient's $SpO_2$ reacts to changes in $O_2$ supplementation. Both are described below.

### Resilience exponent $\alpha$

Throughout hospitalization, a COVID-19 patient experiences declines in $SpO_2$ that may be sustained across a wide range of possible durations. Under normal conditions, there are frequent, momentary drops in $SpO_2$ that are rapidly recovered. The observed frequency of $SpO_2$ drops quickly decays with increasing magnitude, whereby extremely long declines in $SpO_2$ will be observed only very rarely. We speculated that this relationship between the duration of $SpO_2$ declines and their frequency would follow a power-law distribution, $F(x) \propto x^{-\alpha}$ , whereby the frequency with which a decline duration is observed, $F(x)$, is proportional to the duration of the decline, $x$. The exponent $\alpha$ is the scaling parameter – a defining characteristic of a power-law distribution – that quantifies the relative frequency of $SpO_2$ declines across multiple durations. Higher $\alpha$ values reflect fewer long-duration declines and better regulation, whereas lower values suggest more frequent long-duration declines relative to the frequency of shorter declines during hospitalization, i.e., poorer regulation (see *Figure 1*).

Each patient's $SpO_2$ signal was scanned for template patterns representing declines in $SpO_2$ of different durations lasting from 10 minutes up to 2 hours in 5-minute increments (a total of 23 template patterns) *(Figure 1A)*. The number of template matches found for a given duration were then plotted as a function of the duration *(Figure 1B)*. The slope of the resulting line of best fit on a log-log scale is called the resilience exponent $\alpha$ *(Figure 1C)* and gives information about the relative frequency of $SpO_2$ declines of increasing duration.

The exponent $\alpha$ was calculated from the $SpO_2$ signal for each patient's entire hospitalization. First, the minute-to-minute $SpO_2$ signal was smoothed using Gaussian-weighted moving average with a 10-minute window (MATLAB function 'smoothdata'). Second, a series of kernels, or templates, representing decline events were generated for use in the following convolution step. These kernels were designed as the inversed derivative of a Gaussian distribution with durations ranging from 10 minutes to 2 hours in 5-minute increments (i.e., 10, 15, 20, …, 120 minutes). For illustrative purposes, *Figure S1* shows the templates for 10-, 60-, and 120-minute declines.

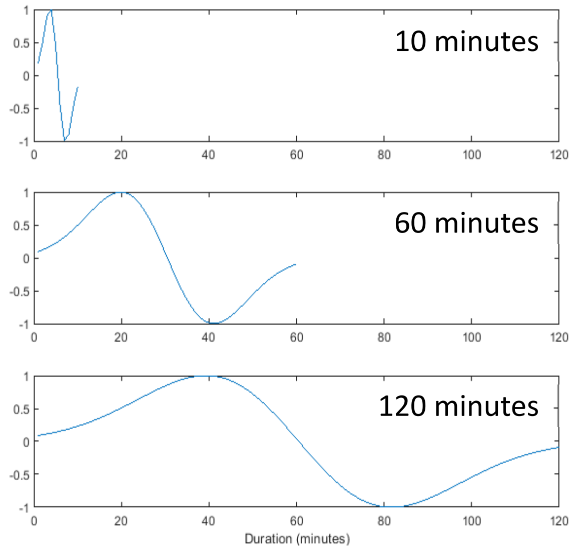


**Figure S1**. Three example "decline" templates used as kernels in convolution with the $SpO_2$ signal.

Third, the $SpO_2$ signal was normalized (between -1 and 1) before performing convolution with each of the 23 kernels (MATLAB function 'conv'). The local minima of the result of convolution were then counted as matches to the decline template using an arbitrary threshold of zero (i.e., all matches were counted). Fourth, the number of matches for each template was plotted against the 23 template durations (i.e., 10 - 120 minutes) for visualization. We speculated that the relationship between the duration of $SpO_2$ declines and their frequency would follow a power-law distribution, $F(x) \propto x^{-\alpha}$ , whereby the frequency with which a decline duration is observed, $F(x)$, is proportional to the duration of the decline, $x$. The scaling relationship can be described by the exponent $\alpha$, which is the slope of the line of best fit when plotted on a log-log scale. Here, instead of using a least squares linear fit, $\alpha$ was calculated using a maximum likelihood estimator,

$$\hat{\alpha} = 1 + n\left[\sum_{i=1}^{n} \ln \frac{x_i}{x_{min}}\right]^{-1},$$

where $x_i$ is the observed duration and $x_{min}$ is the shortest observed duration (i.e., 10 minutes). More information on the power-law distribution and the estimation of the exponent $\alpha$ can be found in (1).

## $O_2$ challenge response

Adjustments to patient $O_2$ supplementation challenge their $SpO_2$ homeostasis and can therefore be used to assess resilience. Under the stimulus-response paradigm, a particular parameter of a (sub-)system (e.g., blood pressure) is probed using a standardized stimulus (e.g., an orthostatic challenge) and the dynamic response is closely observed over time. The degree to which the targeted parameter resists and recovers from perturbation reflects the resilience of the (sub-)system (2).

Here, the changes in $O_2$ supplementation were considered as semi-standardized perturbations, whereby more favorable responses in $SpO_2$ level are also indicative of resilience.

The change in $SpO_2$ ($\Delta SpO_2$) in response to oxygen supplementation challenge was calculated by taking the mean difference of the $SpO_2$ in the 30-minute window immediately before and after the time of the $O_2$ change as noted by the clinical staff in the patient record (*Figure 2*). The number of oxygen adjustments during hospitalization varied between patients. Among those that had two or more changes in oxygen supplementation, the mean $\Delta SpO_2$ and the number of changes necessary were considered as indicators of patient resilience. If the patient only had one change, this single $\Delta SpO_2$ value was used in place of the mean.

## References


1. Clauset A, Shalizi CR, Newman MEJ: Power-Law Distributions in Empirical Data. *SIAM Review* 2009; 51:661–703
2. Varadhan R, Seplaki CL, Xue QL, et al: Stimulus-response paradigm for characterizing the loss of resilience in homeostatic regulation associated with frailty. *Mech Ageing Dev* 2008; 129(11):666-670

## STROBE Checklist

STROBE Statement—Checklist of items that should be included in reports of ***cross-sectional studies***

| | Item No | Recommendation | Page no. | Relevant text |
|---|---|---|---|---|
| **Title and abstract** | 1 | (*a*) Indicate the study's design with a commonly used term in the title or the abstract | 1 | Title page |
| | | (*b*) Provide in the abstract an informative and balanced summary of what was done and what was found | 2 | Abstract |
| **Introduction** | | | | |
| Background/rationale | 2 | Explain the scientific background and rationale for the investigation being reported | 4-5 | Introduction |
| Objectives | 3 | State specific objectives, including any prespecified hypotheses | 4-5 | Introduction |
| **Methods** | | | | |
| Study design | 4 | Present key elements of study design early in the paper | 5-7 | Methods |
| Setting | 5 | Describe the setting, locations, and relevant dates, including periods of recruitment, exposure, follow-up, and data collection | 5-7 | Methods |
| Participants | 6 | (*a*) Give the eligibility criteria, and the sources and methods of selection of participants | 5-6 | Methods |
| Variables | 7 | Clearly define all outcomes, exposures, predictors, potential confounders, and effect modifiers. Give diagnostic criteria, if applicable | 6-7 | Methods |
| Data sources/ measurement | 8* | For each variable of interest, give sources of data and details of methods of assessment (measurement). Describe comparability of assessment methods if there is more than one group | 6-7 | Methods |
| Bias | 9 | Describe any efforts to address potential sources of bias | 6-7 | Methods |
| Study size | 10 | Explain how the study size was arrived at | 5 | Methods |
| Quantitative variables | 11 | Explain how quantitative variables were handled in the analyses. If applicable, describe which groupings were chosen and why | 6-7 | Methods |
| Statistical methods | 12 | (*a*) Describe all statistical methods, including those used to control for confounding | 7 | Methods |
| | | (*b*) Describe any methods used to examine subgroups and interactions | N/A | |

| | | | | |
|---|---|---|---|---|
| | | (*c*) Explain how missing data were addressed | N/A | |
| | | (*d*) If applicable, describe analytical methods taking account of sampling strategy | N/A | |
| | | (*e*) Describe any sensitivity analyses | N/A | |
| **Results** | | | | |
| Participants | 13* | (a) Report numbers of individuals at each stage of study—eg numbers potentially eligible, examined for eligibility, confirmed eligible, included in the study, completing follow-up, and analysed | N/A | |
| | | (b) Give reasons for non-participation at each stage | N/A | |
| | | (c) Consider use of a flow diagram | N/A | |
| Descriptive data | 14* | (a) Give characteristics of study participants (eg demographic, clinical, social) and information on exposures and potential confounders | 8 | Results |
| | | (b) Indicate number of participants with missing data for each variable of interest | N/A | |
| Outcome data | 15* | Report numbers of outcome events or summary measures | 8 | Results |
| Main results | 16 | (*a*) Give unadjusted estimates and, if applicable, confounder-adjusted estimates and their precision (eg, 95% confidence interval). Make clear which confounders were adjusted for and why they were included | 8-9 | Results |
| | | (*b*) Report category boundaries when continuous variables were categorized | N/A | |
| | | (*c*) If relevant, consider translating estimates of relative risk into absolute risk for a meaningful time period | N/A | |
| Other analyses | 17 | Report other analyses done—eg analyses of subgroups and interactions, and sensitivity analyses | N/A | |
| **Discussion** | | | | |
| Key results | 18 | Summarise key results with reference to study objectives | 9-11 | Discussion |

| | | | | |
|---|---|---|---|---|
| Limitations | 19 | Discuss limitations of the study, taking into account sources of potential bias or imprecision. Discuss both direction and magnitude of any potential bias | 12-13 | Discussion |
| Interpretation | 20 | Give a cautious overall interpretation of results considering objectives, limitations, multiplicity of analyses, results from similar studies, and other relevant evidence | 13 | Conclusion |
| Generalisability | 21 | Discuss the generalisability (external validity) of the study results | 12 | Discussion |
| **Other information** | | | | |
| Funding | 22 | Give the source of funding and the role of the funders for the present study and, if applicable, for the original study on which the present article is based | N/A | |

*Give information separately for exposed and unexposed groups.

**Note:** An Explanation and Elaboration article discusses each checklist item and gives methodological background and published examples of transparent reporting. The STROBE checklist is best used in conjunction with this article (freely available on the Web sites of PLoS Medicine at http://www.plosmedicine.org/, Annals of Internal Medicine at http://www.annals.org/, and Epidemiology at http://www.epidem.com/). Information on the STROBE Initiative is available at www.strobe-statement.org.
